# Genetic Transformation of an Obligate Anaerobe, *P. gingivalis* for FMN-Green Fluorescent Protein Expression in Studying Host-Microbe Interaction

**DOI:** 10.1371/journal.pone.0018499

**Published:** 2011-04-15

**Authors:** Chul Hee Choi, Jefferson V. DeGuzman, Richard J. Lamont, Özlem Yilmaz

**Affiliations:** 1 Department of Periodontology, University of Florida, Gainesville, Florida, United States of America; 2 Center for Oral Health and Systemic Disease, University of Louisville, Louisville, Kentucky, United States of America; 3 Emerging Pathogens Institute, University of Florida, Gainesville, Florida, United States of America; Institut de Pharmacologie et de Biologie Structurale, France

## Abstract

The recent introduction of “oxygen-independent” flavin mononucleotide (FMN)-based fluorescent proteins (FbFPs) is of major interest to both eukaryotic and prokaryotic microbial biologists. Accordingly, we demonstrate for the first time that an obligate anaerobe, the successful opportunistic pathogen of the oral cavity, *Porphyromonas gingivalis,* can be genetically engineered for expression of the non-toxic green FbFP. The resulting transformants are functional for studying dynamic bacterial processes in living host cells. The visualization of the transformed *P. gingivalis* (PgFbFP) revealed strong fluorescence that reached a maximum emission at 495 nm as determined by fluorescence microscopy and spectrofluorometry. Human primary gingival epithelial cells (GECs) were infected with PgFbFP and the bacterial invasion of host cells was analyzed by a quantitative fluorescence microscopy and antibiotic protection assays. The results showed similar levels of intracellular bacteria for both wild type and PgFbFP strains. In conjunction with organelle specific fluorescent dyes, utilization of the transformed strain provided direct and accurate determination of the live/metabolically active *P. gingivalis*' trafficking in the GECs over time. Furthermore, the GECs were co-infected with PgFbFP and the ATP-dependent *Clp* serine protease-deficient mutant (*ClpP-*) to study the differential fates of the two strains within the same host cells. Quantitative co-localization analyses displayed the intracellular PgFbFP significantly associated with the endoplasmic reticulum network, whereas the majority of *ClpP-* organisms trafficked into the lysosomes. Hence, we have developed a novel and reliable method to characterize live host cell-microbe interactions and demonstrated the adaptability of FMN-green fluorescent protein for studying persistent host infections induced by obligate anaerobic organisms.

## Introduction

The advent of the green-fluorescent protein (GFP) technology generated a myriad of *in-vivo* and *in-vitro* cell imaging applications including spatio-temporal analysis and expression of diverse cell signaling molecules, cellular organelles, and the high-throughput functional annotation of genome sequences in various organisms [1], [2], [3]. Also, the GFP protein and its variants have been successfully used to analyze the prokaryotic organisms, such as bacterial pathogens, and their interactions with various host cells and the cellular machineries [4]. While the GFP-based biosensors revolutionized the imaging techniques, their strict requirement for molecular oxygen as a co-factor for the synthesis of their chromophores significantly limited their applications for anaerobic microorganisms and cellular microenvironments [5].

The recent discovery of oxygen-independent flavin mononucleotide (FMN)-based fluorescent proteins (FbFPs), engineered from the blue-light photoreceptors of *Bacillus subtilis* and *Pseudomonas putida,* opened up an exciting avenue of research for real-time imaging of live anaerobic microorganisms and anaerobic inter-and intracellular processes [6]. Drepper at al. showed that the codon optimization of these photoreceptors in the light oxygen voltage domains of *E. coli* and the “facultative” anaerobic bacterium *Rhodobacter capsulatus* generates significant degree of cyan-green fluorescence expression both in the absence and presence of oxygen. Subsequently, another study illustrated that the FbFPs can be also used as reporter proteins for fungi such as *Saccharomyces cerevisiae* and the pathogen *C. albicans* under hypoxic conditions [7]. Recent reports further indicate the versatile applications of these non-toxic anaerobically fluorescent proteins for performing quantitative bacterial real-time assays in addition to examining metabolic activities of marine bacterial species (*Roseobacter* and *Phaeobacter spp*.) under various conditions [8].

*Porphyromonas gingivalis* is a successful opportunistic pathogen of the oral mucosa and prominent member of the oral biofilms. Host-pathogen interactions involving the host-adapted pathogen, *P. gingivalis* have been studied extensively in various cell types and animal models [9], [10], [11], [12], [13], [14], [15], [16]. Nevertheless the precise characterization of the intracellular trafficking and the ultimate fate of *P. gingivalis* in host cells including its preferential host cell type, gingival epithelial cells, remain incomplete. This has been to a great extent due to the lack of genetic molecular tools for imaging of the live/metabolically active anaerobic microorganisms. We demonstrate here that the obligate anaerobe, *P. gingivalis*, can be genetically altered for FMN fluorescent protein expression. *P. gingivalis*-FbFP transformants (PgFbFP) were made by utilizing the FMN-based fluorescent Bs2 protein expression system [6]. The transformants produced bright green-fluorescence and biosynthesis was independent of oxygen. Our functional assays with the PgFbFP demonstrated that this new genetic tool enables the direct examination of metabolically active *P. gingivalis* infectious processes under oxygen-limited or strictly anaerobic biological systems. The green-fluorescent transformants were used in conjunction with the lysosomal and endoplasmic reticulum (ER) specific fluorescent markers. Our results showed that we can distinctly determine *P. gingivalis* co-localization with the specific cellular compartments (e.g. lysosomal versus ER) in primary GECs. Since our earlier study indicated that the ATP-dependent Clp serine protease (ClpP) of *P. gingivalis* is likely to be critical for the organism's optimum adaptation to the intracellular life and survival in oral epithelial cells [17], GECs were infected with PgFbFP and the isogenic *ClpP-* mutant of the wild strain simultaneously. The co-infection of the host cells with the green fluorescent organism and its non-fluorescent mutant (detected by the anti-*P. gingivalis*-specific polyclonal antibody coupled with blue fluorescent conjugated secondary antibody) provided initial characterization on the cellular machineries differentially involved in the regulation of the *P. gingivalis*' trafficking.

The non-toxic fluorescent biosensor technology is a significant, developing field and it is of great interest to biologists for the study of a variety of different biological processes. Undoubtedly, the construction of an oxygen-independent fluorescent protein expression in strict anaerobic organisms such as *P. gingivalis* can provide numerous analysis platforms for studying mechanisms/dynamics of chronic infections in variety of host cells and systems.

## Results

### Fluorescence Validation Assays for the FbFP Expression in *P. gingivalis* Transformant

**Figure 1:**
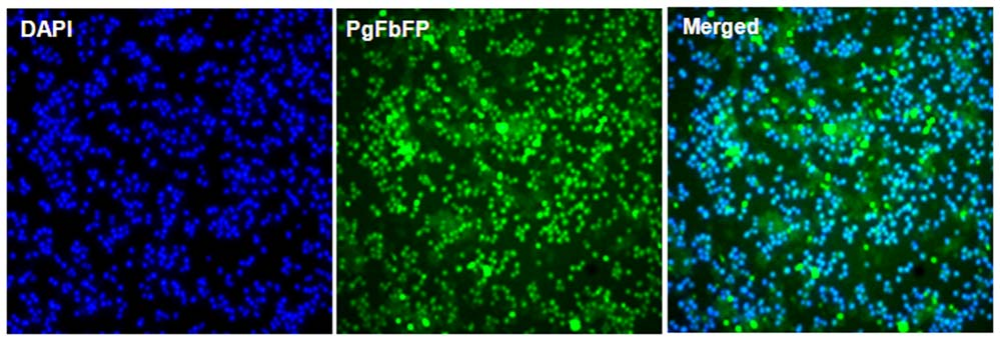
Visualization of the FMN-fluorescent protein expression in transformed *P. gingivalis.* Anaerobically grown transformants were fixed onto the glass slides and examined by epifluorescence microscopy. The samples displayed bright green fluorescence confirming the strong uniform expression of the FbFPs in the *P. gingivalis* cells. DAPI (blue) was used for staining of bacterial nucleic acids. The sequentially acquired images in green and blue channels were merged into a single image (Magnification of 400X).

*P. gingivalis* cells were genetically altered via conjugation of the recombinant *P. gingivalis-E. coli* shuttle vector, pCJO1 (derived from pT-COW) containing FbFP insert from Bs2-evoglow expression plasmid. The resulting transformants, grown anaerobically, were examined for the FbFP expression by epifluorescence microscopy (Fig. 1). Bright green fluorescence levels were visualized in the PgFbFP cells (Fig.1) while the wild-type *P. gingivalis* cells did not produce any detectable fluorescence (not shown). DAPI nucleic acid staining was used to confirm the bacterial cells in the samples (Fig. 1). Thus, fluorescence microscopy confirmed a high degree of accumulation of FbFP in the PgFbFP cells which exhibited a large uniform amount of brightness (Fig. 1). The epi-illumination produced slight axial diffraction (fluorescence flare) in some of the cells due to the fluorescent light from above and below the plane of focus.

**Figure 2:**
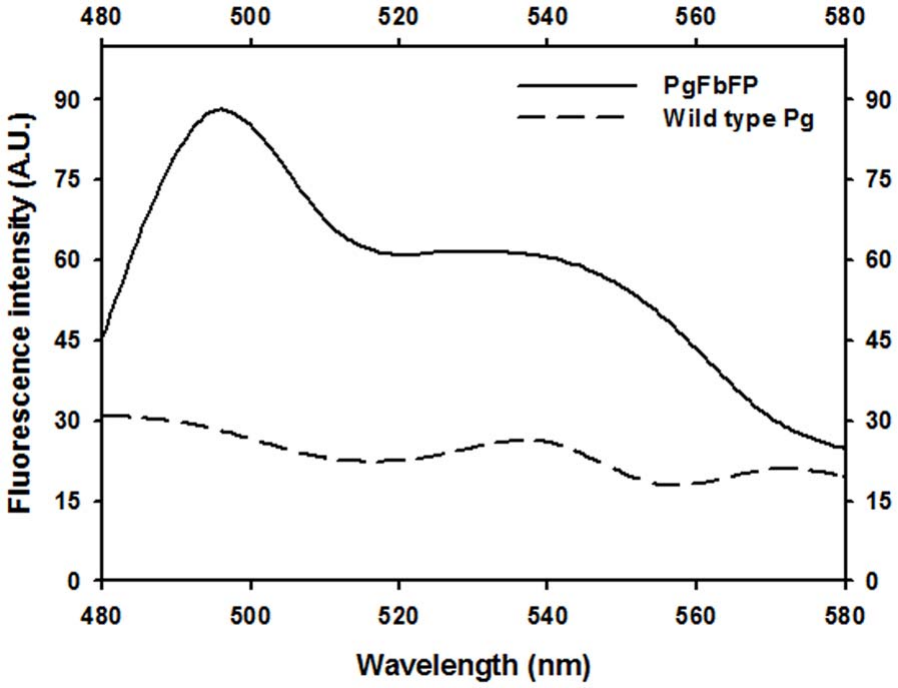
Fluorescence analysis of PgFbFP. The fluorescence emission spectra and the intensities of the *P. gingivalis* strains were analyzed at an excitation wavelength of 450 nm. High fluorescence levels were obtained for the transformant and the maximum emission was observed around 500 nm. The fluorescence intensities represent averages of three independent measurements.

In parallel experiments, we also measured the fluorescence intensities of PgFbFP and wild-type *P. gingivalis* using a spectrofluorometer at excitation wavelength, 450 nm (Fig. 2). The analysis of the bacterial samples further confirmed that the transformant bacteria exhibited strong levels of brightness (a maximal fluorescence emission at 495 nm upon excitation with the blue light) similar to the levels previously reported for the FbFP expression in *E. coli* and *C. albicans* [7]. There was no fluorescence observed in the wild type (Fig. 2). Hence, these analyses established the adaptability of FbFP expression system in an obligate anaerobe that is a member of the human oral microbiome [18].

### Examination of the *In-vitro* Growth and Host Cell Invasion by *P. gingivalis* Transformant

**Figure 3:**
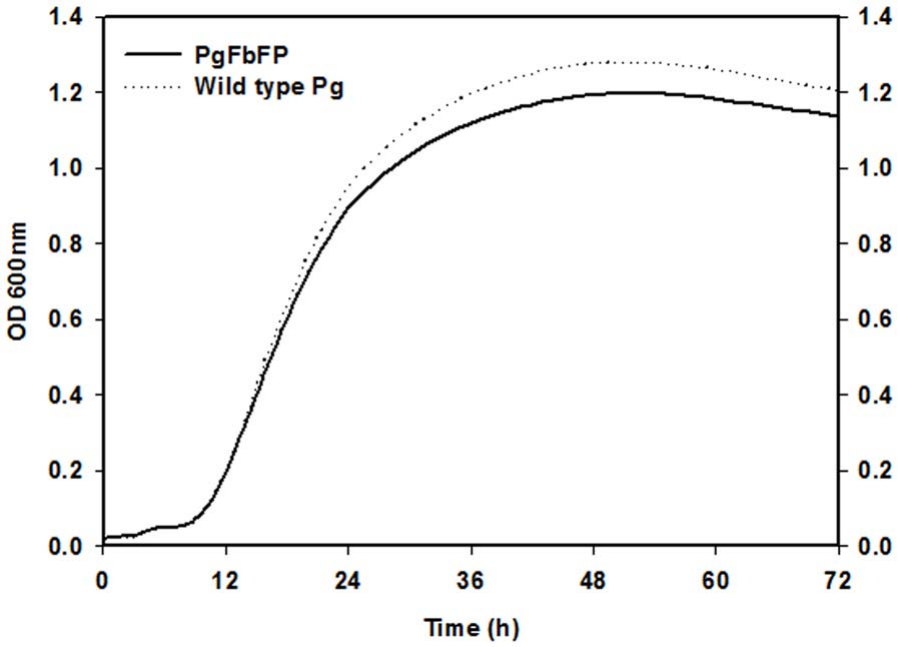
Growth measurements of PgFbFP. Transformed bacteria were grown in defined liquid *P. gingivalis* (TSB) media at 37°C anaerobically for 72 h (late logarithmic phase). The optical density analyses of both wild and the transformant bacteria revealed similar growth dynamics demonstrating the stable metabolism of PgFbFP cells. Data are representative of at least three separate measurements performed in duplicate.

Prior to performing functional host cell infection assays, we studied the *in-vitro* growth dynamics of PgFbFP in *P. gingivalis*' defined TSB liquid media [19]. PgFbFP was inoculated into the anaerobically pre-conditioned TSB media and cultured anaerobically at 37°C for 72 h (late-exponential phase). The results showed that the PgFbFP and the wild type *P. gingivalis* had similar growth rates indicating that the genetic manipulation of the organism for the FbFP expression did not affect its basic growth/cellular metabolism (Fig. 3).

**Figure 4:**
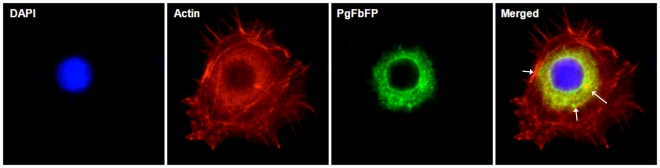
Imaging of PgFbFP invasion of GECs by fluorescence microscopy. Epifluorescent visualization of a representative field of intracellular PgFbFP (green) at 24 h post-infection in GECs revealed high levels of viable bacteria. Actin cytoskeleton was stained by phalloidin-TRITC (red) and nuclei stained with DAPI (blue) to outline the bacterial localization in the host cells. The merged image displays prominent localization of PgFbFPs with the highly polymerized actin filaments and globular actin cytoskeleton around the nucleus (yellow). White arrows point to this dynamic interaction.

Next, we wanted to assess whether PgFbFP can efficiently invade and proliferate in the primary GECs as the wild type. The antibiotic protection assays were performed as described before [20]. Infection by both PgFbFP and wild type resulted similar numbers of intracellular bacteria at 24 h post-infection, confirming that the GEC invasion efficiency and intracellular survival level were similar for both strains (data not shown). Subsequently, PgFbFP infection of GECs at 12 and 24 h was visualized by fluorescence microscopy as we described previously [21]. In agreement with the antibiotic protection assay, visualization of the infection of the GECs by the transformed bacteria by fluorescence imaging displayed a high level of cellular infection (Fig. 4). Quantitative analysis of the bacterial fluorescence by NIH ImageJ analysis software indicated that at 24 h infected cells harbored at least 2.5 times more bacteria than at 12 h (data not shown). This was consistent with the ability of wild *P. gingivalis* to successfully proliferate in GECs over time [22]. Thus, the data further verified that PgFbFP can effectively invade, survive, and replicate in the GECs, which are the central cell type for successful colonization of *P. gingivalis* in the oral cavity.

### Analyses of *P. gingivalis* Trafficking in the Primary GECs through FbFP Expressing Transformant

*P. gingivalis* is a host-adapted pathogen that can successfully survive and proliferate in cultures of primary GECs for extended periods of time, and later spread intercellularly [21], [23], [24]. In line with this, the infection can modulate and interfere with a variety of GEC responses [25], [26], [27], [28], [29], [30].

Bacterial intracellular trafficking studies have shown that ER structures are frequently utilized by persistent organisms to promote their intracellular growth due to the nutritionally rich nature of these components [31], [32]. *P. gingivalis* is localized to the perinuclear region of the host cells, however it has not been determined whether the intracellular organism associates with any subcellular compartments in order to further its intracellular life in GECs [14], [22].

**Figure 5:**
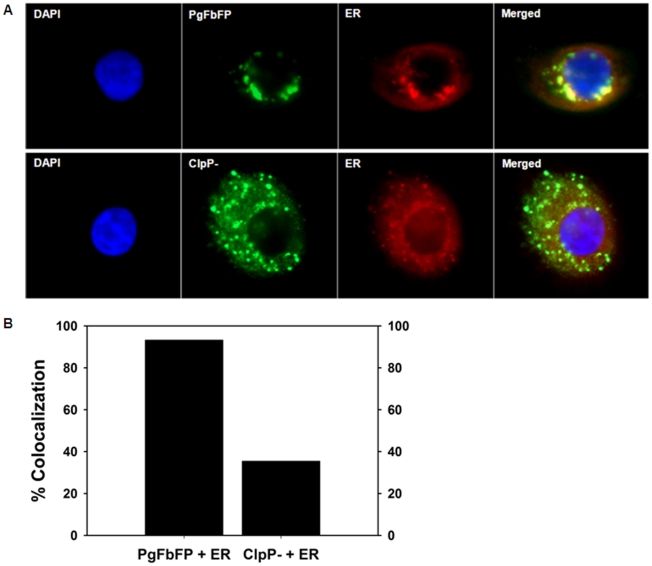
Analysis of *P. gingivalis* association with the ER network. **A.** GECs were infected with PgFbFP (expressing green fluorescent, upper panel) or *ClpP*- mutant (detected by anti-*P. gingivalis* antibody followed by green fluorescent secondary antibody, lower panel), incubated with the ER Tracker (red), and visualized by fluorescence microscopy. The images are representative of at least two separate experiments performed in duplicate. **B.** Quantitative co-localization of *P. gingivalis* with the ER. Images of cells obtained from the GECs infected with PgFbFP or *ClpP*- mutant as described in A were analyzed for co-localization with the ER using Mander's coefficient correlation by JACoP/ImageJ analysis software. The result is representative of an average of 50 cells per sample studied from at least two separate experiments performed in duplicate.

As we have developed a tool that can unequivocally follow live *P. gingivalis* inside host cells, GECs were infected with PgFbFP (green) and stained with the ER- specific fluorescent dye (ERTracker-Red) to determine the potential co-localization between ER and the intracellular bacteria (Fig. 5A and B). Fluorescence microscopy analysis of the samples demonstrated a significant level of overlap between the PgFbFP strain and the ER structures, which are located in the perinuclear compartments typical of the ER (Fig. 5A). The co-localization events were quantified by the JACoP toolbox under NIH ImageJ using Manders' coefficient correlation [33]. This object-based fluorescent intensity analysis revealed ∼90% co-localization rate between the *P. gingivalis* and ER networks in the GECs (Fig. 5B). In contrast, as a control, we infected GECs with a *ClpP-* mutant of *P. gingivalis* and studied the co-localization events between the mutant bacteria and the ER. The mutant bacterial localization was detected by anti-*P. gingivalis*-specific polyclonal antibodies coupled with green fluorescent conjugated secondary antibody. As shown in Fig 5A, The *ClpP-* localization did not significantly overlap with the ER network (Fig. 5A). Measurement of the co-localization rate by the Manders' coefficient correlation analysis showed a ∼34% association, indicating only a partial co-localization (Fig. 5B).

**Figure 6:**
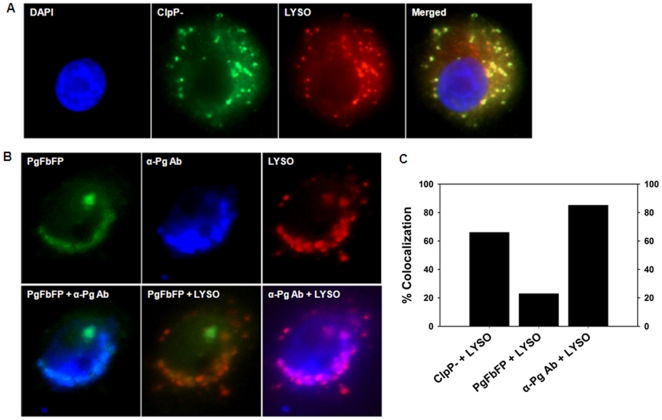
Analysis of *ClpP*- mutant association with lysosomes. **A.** GECs were infected with the *ClpP*- mutant of *P. gingivalis* and incubated with the Lyso Tracker (red). The *ClpP*-mutant strain was detected by green fluorescent antibody. Double staining analysis of the obtained images indicated a large level of co-localization (yellow) between the mutant and the lysosomes. **B.** Distinct intracellular trafficking of *P. gingivalis* strains in GECs. GECs were co-infected with PgFbFP (green) and *ClpP*- mutant simultaneously, and incubated with the Lyso Tracker (red). The samples were reacted with the anti-*P. gingivalis* antibody coupled with blue fluorescent secondary antibody which labeled both PgFbFP (green) and *ClpP*- mutant, blue (upper panel). The overlay images of PgFbFP (green) and the anti-*P. gingivalis* staining (blue) displayed the differentially localized strains in the same cell (yellow for the PgFbFP staining and clean blue for *ClpP*- mutant). Image analysis showed no significant co-localization (yellow) between PgFbFP (green) and lysosomes (red), whereas the merged images of lysosomes (red) and anti-*P.gingivalis* staining (blue) produced a significant level of purple (co-localization of *ClpP*- mutant with lysosomes). **C.** Quantitative analyses of *P. gingivalis* co-localization with lysosomes. The co-localization assays depicted in Figure 6A and B were analyzed quantitatively using Mander's coefficient correlation by JACoP/ImageJ analysis software. The results are representative of an average of 50 cells per sample studied from at least two separate experiments performed in duplicate.

Our previous study and the studies on *Listeria spp,.* indicated the ClpP expression is essential for the intracellular survival [17], [34]. Therefore, *P. gingivalis ClpP-*infected cells were stained with the lysosome specific fluorescent dye (LysoTracker-Red) and post-labeled with the anti- *P. gingivalis* antibody coupled with fluorescent conjugated secondary antibody (green) to determine potential subcellular localization of the mutant cells with lysosomes at 24 h infection. (Fig. 6A). The collected images produced an extensive degree of yellow hot- spots (co-localization) and quantitative analysis measured ∼66% co-localization between the lysosomal structures and the mutant bacteria (Fig. 6C). The results suggested the majority of mutant bacteria trafficked into the lysosomes and potentially were in the process of lysosomal degradation.

One of the major limitations of antibody based bacterial detection techniques for intracellular trafficking/fate determination studies is that antibody staining cannot distinguish damaged or dead bacteria from live bacteria and/or bacterial antigens from intact/metabolically active versus degrading microorganisms. Therefore, we developed a novel approach whereby we co-infected GECs with the genetically modified “green fluorescent *P. gingivalis*” and the *ClpP-* simultaneously to analyze the subcellular compartmentalization of *P. gingivalis* and its isogenic mutant in the same host cell throughout the infection by triple fluorescent staining. The non-fluorescent mutant (*ClpP-*) was detected with the anti-*P. gingivalis* antibodies as described above, and the lysosomes were stained with LysoTracker-Red, as shown in Fig. 6B. Quantitative co-localization analysis of the two *P. gingivalis* strains utilizing the specific organelle dye within the same host cells further verified the ER network as a prominent subcellular niche for live *P. gingivalis* infection in GECs, while the majority of *ClpP-* mutants trafficked into the lysosomes at 24 h post-infection (Fig. 6B and C).

## Discussion

Within the last decade, genetically encoded fluorescent molecule-based technologies have become widely used approaches for the study of host cell-parasite interaction [35]. Nevertheless, the absence of oxygen-independent fluorescent protein systems significantly restricted the direct examinations of infectious agents that require limited or strict anaerobic living conditions.

*P. gingivalis,* a gram-negative strict anaerobe and successful colonizer of oral tissues, can survive, replicate, and spread in GECs [18], [36]. Since the organism can be cultured *in-vitro* and is a prominent member of oral biofilms, *P. gingivalis* is considered a facultative intracellular pathogen. However, it can rather be viewed essentially as an intracellular pathogen if one considers that human epithelial cells are the privileged niche for multiplication, and human oral mucosa as the pathogen's primary reservoir [13], [18]. In the absence of fluorescent-based genetic tools only limited details of the *P. gingivalis* infectious cycle and the interacting host molecules in the oral tissues have emerged.

Several approaches have been used in the past for imaging of anaerobic microorganisms such as *P. gingivalis* in *ex-vivo or in-vitro* host models [21], [37], [38], [39]. These techniques frequently involved specific polyclonal antibodies or 16S rDNA probes which cannot distinguish live organisms from dead or damaged/fragmented organisms, or using of non-specific chloromethyl derivative fluorescent dyes which are significantly diluted after a couple of generations.

This study demonstrates for the first time the versatility of the recently reported oxygen-independent and non-toxic FbFPs technology for studying real-time host cell-anaerobic bacteria interaction. The successful transformation of *P. gingivalis* into a green fluorescent bacterium with a healthy basic metabolism and consistent phenotype for the invasion, multiplication and survival in the GECs were key findings for a variety of future analyses. Moreover, we developed a novel method for an accurate qualitative (visual) and quantitative evaluation of the differentially stained bacteria within the same cells (Fig. 6B and C). This provided a reliable spatial comparative analysis of two different strains of *P. gingivalis* and their relationship to a specific organelle (e.g. lysosomes, ER), and further validated the distinct fates of *P. gingivalis* and its isogenic *ClpP*- mutant in the primary GECs following 24 h infection. These new findings are consistent with the organism's previously described pro-survival phenotype in the GECs [23], [24], [30]. Also, the preliminary finding of the *ClpP*- mutant trafficking to the lysosomal pathway substantiates the importance of the stress-induced protease, ClpP, for the intracellular survival of *P. gingivalis* in GECs [17]. This result is also in agreement with the results obtained by *Listeria monocytogenes* where ClpP serine protease induces the early escape of the pathogen from the phagosomal compartments [34], [40].

While this is an initial study in the direct characterization of metabolically active *P. gingivalis* trafficking in the human gingival cells, it certainly offers more detailed analyses of diverse inter- and intracellular bacterial trafficking and infectious processes induced by the organism not only in the GECs but also in other host cell types. Accordingly, the FbFP technology can be effectively utilized for long-term live cell bacterial imaging in the host cells including in animal models which are widely utilized by the researchers studying the local and systemic implications of chronic infections by opportunistic anaerobic pathogens.

## Materials and Methods

### Bacteria, Growth Conditions, and Construction of PgFbFP

*P. gingivalis* ATCC *33277*, ATP-dependent Clp serine protease-deficient isogenic mutant (*ClpP-*), and PgFbFP were cultured anaerobically at 37°C in trypticase soy broth (TSB) supplemented with yeast extract (1 mg ml-1), haemin (5 µg ml-1) and menadione (1 µg ml-1). Erythromycin (10 ug ml-1) was added to the media for culture of the mutant strain [17]. The media for PgFbFP strain was supplemented with tetracycline (3 µg ml-1). All bacteria were grown for 24 h, harvested by centrifugation at 6000 g and 4°C for 10 min, washed twice, and resuspended in Dulbecco's Phosphate-buffered saline (Sigma) pH 7.3 before they were reacted with host cells. The number of bacteria was determined using a Klett-Summerson photometer.

#### Construction of PgFbFP Strain with pCJO1

The evoglow-Bs2plasmid from the Evoglow kit (Evocatal, Germany) was used to amplify a flavin mononucleotide (FMN)-based fluorescent gene to be fused with the *P. gingivalis* (ATCC 33277) *fimA* promoter region (Fig S1 and Table S1) [41]. The fusion PCR protocol included an initial step of 95°C for 5 min, denaturation step of 95°C for 1 min, annealing step of 60°C for 1 min, extension step of 72°C for 1 min, and a final elongation step of 72°C for 10 min (Fig S2). The fusion PCR product was then cloned into the Sal1 and SPH1 sites of *P. gingivalis-E. coli* shuttle vector, pT-COW [42] and transformed into *E. coli* S17 cells. The recombinant plasmid (named pCJO1) in the *E. coli* S17 donor cells were subsequently transferred to *P. gingivalis* through conjugation [28]. Finally, the tetracycline-resistant *P. gingivalis* clones were grown agar plates and selected for fluorescence analyses.

### Generation and Culture of Human Primary Gingival Epithelial Cells

Primary cultures of GECs were generated as described previously [20]. No subject recruitment per se was done. Adult patients were selected at random and anonymously from those presenting at the University of Florida Dental Clinics for tooth crown lengthening or impacted third molar extraction. Gingival tissue that would otherwise be discarded was collected after informed written consent by the patient (approved by the Institutional Review Board of University of Florida). No patient information was collected. This study is approved by the Institutional Review Board under the University of Florida human subjects assurance number FWA 00005790. Briefly, healthy gingival tissue was obtained after oral surgery, and surface epithelium was separated by overnight incubation with 0.4% dispase. Cells were cultured as monolayers in serum-free keratinocyte growth medium (KGM) (Lonza) at 37°C in 5% CO_2_. GECs were used for experimentation at 75–80% confluence and cultured at least for 24 h before infection with bacterial cells.

### Fluorescence Assays for the Analyses of PgFbFP

FbFP-expressed *P. gingivalis* strain grown anaerobically at 37°C were washed with PBS, placed on microscopic glass slides and fixed with 10% neutral buffered formalin for 15 min at room temperature. The samples were treated with 4,6-diamidino-2-phenylindole (DAPI) (1 µg/ml) (Sigma) to visualize the bacterial nucleic acids. Finally, the PgFbFP samples were washed twice with PBS and analyzed using a Zeiss Axio imager A1 epifluorescence microscope equipped with optical filter sets with excitation, 475/40; emission, 500/10 for the green fluorescence, and excitation 360; emission 440/50 nm for the DAPI. The images were collected by a cooled CCD camera (Qimaging). Single exposure images were captured sequentially and saved by Qcapture software as we described previously [26].

Fluorescence emission spectra of the PgFbFP cells were measured with a BioTek Instrument, model Synergy MX Multi-mode spectrometer (excitation wavelength, 450 nm) and analyzed by Software: Gen5. Fluorescence intensity is expressed in arbitrary units (A.U.).

### Growth Measurement of PgFbFP

*P. gingivalis* ATCC *33277* and the PgFbFP strain were cultured anaerobically at 37°C in the liquid broth media (TSB) as explained above. Fifty microliters of overnight cultures were used to inoculate in 10 ml of TSB medium pre-incubated anaerobically at 37°C. Bacteria were further incubated for 3 h, 6 h, 12 h, 24 h, 48 h, and 72 h. At each incubation time point, OD600 were measured using a BioMate 3 Spectrophotometer (Thermo Electron Corporation).

### Epithelial Cell Invasion Assays

#### Antibiotic Protection Assay

PgFbFP invasion of GECs was determined by the antibiotic protection assay described previously [20]. In brief, bacteria in KGM were incubated with GECs in 6-well plates for 60 min at 37°C. After washing with PBS, remaining external bacteria were killed with metronidazole (200 µg ml-1) and gentamicin (300 µg ml-1) and further incubated for 24 h. GECs were washed and lysed with sterile distilled water, and intracellular bacteria were enumerated by culture on blood agar supplemented with haemin and menadione.

#### Imaging and Quantitation of the PgFbFP Invasion by Fluorescence Microscopy

GECs were seeded onto 2-well chambered cover-glass slides (Nalge-Nunc International) at a density of 2×10^4^ cells per well and cultured for 24 h. Cells were infected with PgFbFP at a multiplicity of infection (m.o.i.) of 100 at 37°C in 5% CO_2_ incubator. After 12 or 24 h incubation, the slides were washed four times with PBS containing 0.1% Tween 20 to remove the non-adherent bacteria. Cells were fixed in 10% neutral buffered formalin, and rinsed in PBS at room temperature. F-actin was labeled with phalloidin–tetramethylrhodamine B isothiocyanate (TRITC) (Sigma) at 1∶100 for 45 min. The samples were mounted in anti-fade mounting medium with DAPI (Vector Laboratories). Samples were visualized using the fluorescence microscope system described above. Acquired images were analyzed for fluorescence intensity with NIH ImageJ analysis software [25].

### Fluorescence Imaging of Bacterial Trafficking in GECs

GECs cultivated on the 2-well chambered cover-glass slides were infected with *P. gingivalis* wild type, *ClpP-* mutant or PgFbFP at an m.o.i of 100 at 37°C for 24 h. The infected live cells were incubated with ERTracker-Red (1 µM/ml) (Invitrogen), a specific fluorescent cell-permeable dye for endoplasmic reticulum labeling or LysoTracker-Red (1 µM/ml) (Invitrogen), a specific fluorescent cell-permeable dye for lysosomes, for 30 min. Cells were fixed with 10% neutral buffered formalin and wild type and *Clp-* infected cells permeabilized by 0.1% Triton X-100 and reacted with anti-*P. gingivalis* 33277 rabbit polyclonal antibody (1∶1000) followed by Oregon Green 488 goat secondary antibody (1∶500) (Invitrogen). For the co-infection studies, GECs were infected with PgFbFP and *ClpP-* mutant strains simultaneously and the live cells were labeled with LysoTracker-Red and then fixed and permeabilized as explained above. The samples were reacted with anti-*P. gingivalis* 33277 rabbit polyclonal antibody followed by Marina Blue Goat secondary antibody (1∶500) (Invitrogen). The samples were mounted in anti-fade mounting medium (Vector Laboratories) and visualized using the fluorescence microscope system described above.

### Measuring Co-localization Events

Co-localization analysis was carried out using the JACoP tool under NIH ImageJ software as described previously [33]. Images were pre-processed to correct uneven illumination and to minimize noise and background. The co-localization rates were measured based on Manders' coefficient, which varies from 0 to 1. A coefficient value of zero corresponds to non-overlapping images while a value of 1 reflects 100% co-localization between the images being analyzed.

## Supporting Information

Figure S1 The construction of FbFP expressing *P. gingivalis* transformant. The codon usage of *E. coli* FbFP was adapted to *P. gingivalis,* and the resulting gene was placed under the transcriptional control of the *fimA* promoter of *P. gingivalis.*
(DOCX)

Figure S2 *fimA* promoter region and FbFP from pGlOW Bs2 plasmid were amplified by fusion PCR and checked by agarose gel electrophoresis (Fusion 731 bp).
(DOCX)

Table S1 Primer sets used for the fusion PCR amplification.
(DOCX)
