# Supplementary figures and images for: Genetic Transformation of an Obligate Anaerobe, P. gingivalis for FMN-Green Fluorescent Protein Expression in Studying Host-Microbe Interaction
Source: PLoS One. 2011 Apr 15;6(4):e18499. doi: 10.1371/journal.pone.0018499 (PMC3078116; doi:10.1371/journal.pone.0018499)

**
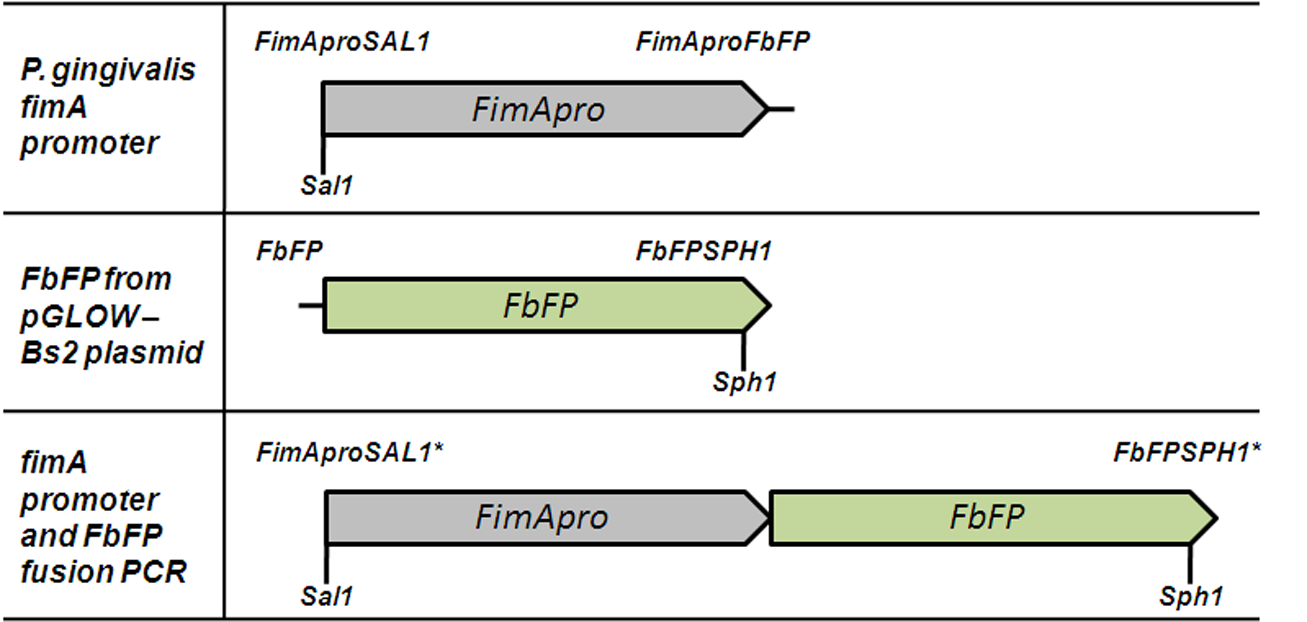
**

Supplement: Figure S1 — The construction of FbFP expressing P. gingivalis transformant. The codon usage of E. coli FbFP was adapted to P. gingivalis, and the resulting gene was placed under the transcriptional control of the fimA promoter of P. gingivalis. (DOCX) [file pone.0018499.s001.docx]

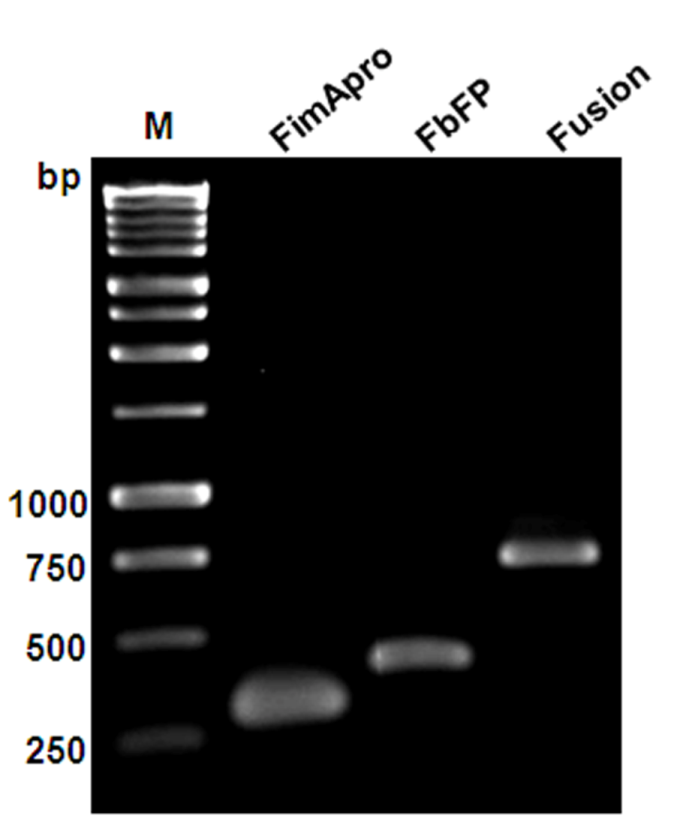

Supplement: Figure S2 — fimA promoter region and FbFP from pGlOW Bs2 plasmid were amplified by fusion PCR and checked by agarose gel electrophoresis (Fusion 731 bp). (DOCX) [file pone.0018499.s002.docx]
