# Supplementary material for: Genetic Transformation of an Obligate Anaerobe, P. gingivalis for FMN-Green Fluorescent Protein Expression in Studying Host-Microbe Interaction
Source: PLoS One. 2011 Apr 15;6(4):e18499. doi: 10.1371/journal.pone.0018499 (PMC3078116; doi:10.1371/journal.pone.0018499)
Supplement: Table S1 — Primer sets used for the fusion PCR amplification. (DOCX) [file pone.0018499.s003.docx]

| **Primers** | **Sequences** | **Products**  **(bp)** |
| --- | --- | --- |
| *FimAproSAL1* | 5’ TGGGTCGACATATGCCTACAGCGAAAAATGG 3’ | 284 |
| *FimAproFbFP* | 5’ GAACGACTGGAACGACGCCATGCTGATGGTGGCATTACCTT 3’ |  |
| *FbFP* | 5’ ATGGCGTCGTTCCAGTCGTT 3’ | 447 |
| *FbFPSPH1* | 5’ ATGGCATGCTCACTCGAGCAGCTTTTCATA 3’ |  |
| *FimAproSAL1** | 5’ GTCGACATATGCCTACAGCGAAAAATGGCTG 3’ | 731 |
| *FbFPSPH1** | 5’ GCATGCTCACTCGAGCAGCTTTTCATA 3’ |  |
